# Off-label evaluation of the BD MAX MDR-TB assay for rapid diagnosis of rifampicin and isoniazid resistance of *Mycobacterium tuberculosis* clinical isolates in a high-volume reference laboratory

**DOI:** 10.1128/jcm.00912-25

**Published:** 2025-09-23

**Authors:** Angela Pires Brandao, Fabiane Maria de Almeida Ferreira, Fernanda Cristina dos Santos Simeao, Lucilaine Ferrazoli, Erica Chimara, Rosângela Siqueira de Oliveira, Juliana Maira Watanabe Pinhata

**Affiliations:** 1 Núcleo de Tuberculose e Micobacterioses, Centro de Bacteriologia, Instituto Adolfo Lutz (IAL) 89119, São Paulo, Brazil; 2 Instituto Oswaldo Cruz, Fundação Oswaldo Cruz (FIOCRUZ) 37903 , Rio de Janeiro, Brazil; The University of North Carolina at Chapel Hill School of Medicine, Chapel Hill, North Caroline, USA

**Keywords:** BD MAX MDR-TB, drug resistance, line probe assay, multidrug-resistant tuberculosis, *Mycobacterium tuberculosis*

## Abstract

Drug-resistant tuberculosis (TB) remains a primary global health concern. Multidrug-resistant TB is defined by resistance to at least rifampicin (RIF) and isoniazid (INH), the two key drugs used in TB treatment. The BD MAX Multi-Drug Resistant Tuberculosis (BD MAX) assay is a fully automated real-time PCR platform recommended by the World Health Organization for the initial diagnosis of TB and RIF and INH resistance (RIF-R and INH-R) directly from pulmonary clinical samples. This study aimed to assess the off-label performance of BD MAX in clinical *M. tuberculosis* complex (MTBC) isolates under routine laboratory conditions. The assay was first validated using non-tuberculous mycobacteria (NTM) and MTBC isolates with known mutations. For real-world validation, it was compared to the GenoType MTBDR*plus* by testing 1,440 clinical isolates prospectively. The BD MAX assay correctly excluded MTBC from all NTM cultures. Among MTBC isolates with known mutations, it identified 19 of 20 RIF-R isolates and 14 of 15 INH-R isolates. In prospective testing, BD MAX achieved 99.6% sensitivity (1,403/1,409), 96.8% specificity (30/31), and 99.5% overall accuracy (1,433/1,440) for MTBC detection. For drug resistance detection, it showed 95.2% (40/42) concordance for RIF, 96.8% (30/31) for INH, and 81.3% (13/16) for MDR when compared to MTBDR*plus*. Discrepancies between MTBDR*plus* and BD MAX included heteroresistant cases and unreportable resistance results by BD MAX due to infrequent mutations or low bacterial load. Overall, this study confirms BD MAX as an accurate and reliable tool for MTBC detection and drug resistance profiling in clinical isolates in high-volume TB laboratories.

**IMPORTANCE:**

This study highlights the importance of the BD MAX Multi-Drug Resistant Tuberculosis assay (BD MAX) applied in clinical isolates for the detection of multidrug-resistant tuberculosis (MDR-TB), i.e., *Mycobacterium tuberculosis* resistance to rifampicin and isoniazid. TB is a global health issue, and drug-resistant TB makes treatment more difficult, favoring transmission and disease amplification. The BD MAX platform offers a faster and more automated way to detect TB and drug resistance. The study showed that BD MAX, applied off-label in clinical isolates, accurately identified TB and resistance to rifampicin and isoniazid, with results comparable to those of the widely used line probe assay. This is significant in a high-volume laboratory because it is more straightforward and more rapid than the line probe assay. BD MAX showed some limitations, especially in detecting rare mutations and in cases of low bacterial levels. Overall, this tool could improve TB care, especially in high-volume laboratories.

## INTRODUCTION

According to the World Health Organization (WHO), in 2023, only 40% of the estimated 400,000 new cases of rifampicin-resistant (RIF-R-) or multidrug-resistant tuberculosis (MDR-TB) were diagnosed and received appropriate drug-resistant TB treatment (1). Timely detection of *Mycobacterium tuberculosis* complex (MTBC) drug resistance is essential for effective TB management (2). WHO recommends universal drug susceptibility testing (DST), starting with rapid RIF resistance testing for all presumptive TB cases (3).

Brazil is among the 30 countries with the highest TB and TB/HIV burdens. In 2023, it reported over 80,000 new TB cases, including 118 cases of RIF-R- or MDR-TB in São Paulo state alone, which accounts for 25% of Brazil’s TB cases (3, 4). Rapid molecular tests, which detect the most common genetic mutations associated with drug resistance, offer high sensitivity and specificity (5, 6), enabling earlier treatment.

In Brazil, the Xpert MTB/RIF Ultra (Cepheid, USA) is widely used for initial detection of RIF-R-TB (7), but its limited throughput and inability to detect isoniazid (INH) monoresistance—present in 8% of global cases (8)—pose challenges. Line probe assays (LPAs), such as GenoType MTBDR*plus* and MTBDR*sl* (Hain Lifescience-Bruker, USA), and phenotypic DST (pDST) using BACTEC MGIT 960 are also used in some states’ reference laboratories (9). However, pDST is slow and requires biosafety infrastructure, while LPAs are labor-intensive and prone to cross-contamination (7). The Xpert MTB-XDR (Cepheid), which identifies resistance to INH and second-line drugs, is not yet in use in the country.

Automated molecular platforms, such as the BD MAX Multi-Drug Resistant Tuberculosis assay (BD MAX MDR-TB) (Becton, Dickinson and Company, USA), address these limitations. Designed for sputum testing, BD MAX detects MTBC-specific sequences and mutations in *rpoB*, *katG*, and the *inhA* promoter region (10). It integrates lysis, DNA extraction, and real-time PCR in a closed system, processing up to 24 samples in up to five hours (11, 12), thereby reducing errors and biosafety risks.

Most studies evaluating the BD MAX system were conducted on clinical samples (11–17), as this test is primarily recommended for a rapid initial diagnosis of TB and drug resistance detection. A multicenter study on raw sputum reported 93% sensitivity and 99% specificity for TB detection, 90% sensitivity and 98% specificity for RIF resistance, and 82% sensitivity and 100% specificity for INH resistance, compared to pDST and/or sequencing (12).

At the Adolfo Lutz Institute’s Núcleo de Tuberculose e Micobacterioses laboratory, which is the reference public health laboratory for São Paulo, over 6,000 MTBC isolates are tested annually using LPAs. Incorporating BD MAX could streamline diagnostics by offering a faster, automated alternative that requires fewer manual steps and physical spaces.

This study aimed to evaluate the off-label performance of the BD MAX MDR-TB assay for MTBC-suggestive clinical isolates in a routine reference laboratory setting, compared to the GenoType MTBDR*plus* LPA.

## MATERIALS AND METHODS

### Retrospective validation of the BD MAX MDR-TB assay

Initially, the BD MAX assay was validated on cultured isolates using the *M. tuberculosis* reference strain H37Rv (ATCC 27294) to determine its limit of detection (LoD), the optimal volume of inactivated isolates to be processed, and the required incubation time with the sample treatment reagent (STR), which is a BD MAX’s kit reagent that reduces MTBC viability. Once these parameters were optimized, the assay was applied to non-tuberculous mycobacteria (NTM) and MTBC clinical isolates with known resistance-associated mutations.

### Culturing, inactivation, and DNA extraction of the isolates

Isolates and *M. tuberculosis* H37Rv stored at −70°C in the laboratory repository were reactivated in Löwenstein-Jensen (LJ) or MGIT at a biosafety level 3 (BSL-3) laboratory for BD MAX validation. The isolates were inactivated for testing in the BD MAX system, since the STR reagent was designed for the pre-treatment of sputum samples and is not effective in inactivating the high number of bacilli typically found in cultures, as it only reduces the viability of MTBC (18). The inactivation was also necessary because the BD MAX equipment was located in an area outside the BSL-3 laboratory. Inactivation was performed as follows: 1 mL of the homogenized growth of the isolates in MGIT, or a loopful of the growth on LJ diluted in 1 mL of ultrapure water, was incubated at 95°C for 20 minutes. To enhance cell lysis for DNA extraction by BD MAX, these heat-inactivated isolates were frozen at −20°C, incubated again at 95°C for 20 minutes (19), and stored at −20°C.

### Assessment of the LoD of the BD MAX MDR-TB assay

BD MAX assay’s LoD on cultured isolates was assessed using *M. tuberculosis* H37Rv grown for 28 days at 35±2°C in LJ. To prepare bacterial suspensions for testing, a 10 µL loopful of strain growth was transferred to a screw-cap glass tube containing 5–10 sterile 3 mm glass beads. After vortexing for 2 minutes to disrupt clumps, 5 mL of sterile distilled water was added and vortexed again for 2 minutes. The tube rested for 30 minutes to allow remaining clumps to settle. The supernatant’s turbidity was adjusted to McFarland 0.5 in a new tube using sterile distilled water, vortexed for 30 seconds, and measured with a densitometer (20).

A 1:100 dilution of the bacterial suspension was prepared in 7H9/OADC broth through two tenfold dilution steps: first, 1 mL of the 0.5 McFarland suspension was added to 9 mL broth (10⁻¹); then, 1 mL of the 10⁻¹ suspension was added to 9 mL broth (10⁻²). Further serial tenfold dilutions were made up to 10⁻⁸ (20). Ten-microliter triplicates of dilutions ranging from 10⁻² to 10⁻⁵ were inoculated onto 7H10 agar plates. The plates were tilted to allow the inoculum to spread evenly along the surface. After 21 days of incubation at 35±2°C, colony growth was assessed. Countable colonies were achieved for the 10⁻³, 10⁻⁴, and 10⁻⁵ dilutions of the H37Rv strain, yielding 9,130, 1,000, and 100 CFU/mL, respectively—values within the expected concentration range (20).

According to the manufacturer’s instructions, the sputum sample and STR mixture should be manually agitated 10 times, incubated at room temperature for 5 minutes, then stirred vigorously 10 more times, and left at room temperature for an additional 25 minutes before being transferred to the equipment. Since we used an H37Rv culture—rather than raw sputum samples, which are more challenging to fluidize—and were evaluating the use of the BD MAX with mycobacterial cultures, we also investigated whether the 25-minute incubation following the initial 5-minute incubation of the inactivated H37Rv culture + STR mixture would impact the performance of the assay. Additionally, considering the high number of isolates received by our laboratory, we also tested whether incubating the mixture of H37Rv culture +STR for 24, 48, and 72 hours would affect the BD MAX performance (Table 1). For this, 1 mL of each inactivated suspension of H37Rv (10^−1^ to 10^−8^) was added to 2 mL of STR, and 2.5 mL of this mixture was tested in the BD MAX.

**TABLE 1:** Results of dilutions and incubation periods of the H37Rv reference strain with STR reagent for the assessment of the limit of detection of the BD MAX MDR-TB assay for off-label detection of *Mycobacterium tuberculosis* clinical isolates^*a*^

H37Rv	Incubation times of *M. tuberculosis*H37Rv + the STR reagent														
Strain	5 minutes			5 minutes + 25 minutes (as for sputum samples)			24 hours			48 hours			72 hours		
dilution	MTBC	RIF	INH	MTBC	RIF	INH	MTBC	RIF	INH	MTBC	RIF	INH	MTBC	RIF	INH
10^−1^	D	ND	ND	D	ND	ND	D	ND	ND	D	ND	ND	D	ND	ND
10^−2^	D	ND	ND	D	ND	ND	D	ND	ND	D	ND	ND	D	D	ND
10^−3^	D	ND	ND	D	ND	ND	D	ND	D *katG*	D	ND	ND	D	ND	ND
10^−4^	D	ND	ND	D	ND	ND	D Low	-	-	D Low	-	-	D	ND	D *katG*
10^−5^	D Low	-	-	D Low	-	-	D Low	-	-	D Low	-	-	D	ND	!INH R UNR
10^−6^	-	-	-	-	-	-	-	-	-	-	-	-	-	-	-
10^−7^	-	-	-	-	-	-	-	-	-	-	-	-	-	-	-
10^−8^	-	-	-	-	-	-	!MTB UNR			-	-	-	-	-	-

^*a*^ INH, isoniazid; RIF, rifampicin; D, detected; D Low, MTBC low positive, i.e., *Mycobacterium tuberculosis* complex DNA detected but resistance metrics not measurable due to low bacterial load; D *katG*, mutation detected on *katG* gene; !MTB UNR, BD MAX error MTB unresolved (no MTBC DNA detected and no sample processing control detected); !INH R UNR, BD MAX error INH resistance unreportable (MTBC DNA detected but INH resistance metrics not measurable, either the *katG* or *inhA* promoter probe failed to give a signal with the other signal presenting as wild-type); ND, not detected (10).

For MTBC detection, the highest dilution with positive results for all H37Rv + STR incubation protocols (5 minutes, 5 minutes + 25 minutes, 24 hours, 48 hours, and 72 hours) was 10^−5^ (Table 1). For drug resistance detection, the highest dilution with valid results was 10^−4^ for the 5 minutes and 5 minutes + 25 minutes protocols. For the 24-hour, 48-hour, and 72-hour incubation protocols, false-positive mutation results were obtained. Therefore, we used the 5-minute incubation protocol for all BD MAX tests due to its faster processing time.

The estimated LoD of the BD MAX for MTBC identification corresponded to ~34 CFU/mL in the 2.5  mL mixture of bacterial suspension and STR used per test. For the detection of drug resistance mutations, the LoD was ~340 CFU/mL. Under these conditions, the BD MAX assay was able to detect ~84 CFU for MTBC identification and ~840 CFU for drug resistance detection.

### Standardization of the inactivated cultured isolates for the BD MAX MDR-TB assay

We receive mycobacterial isolates on solid or MGIT media from 82 TB laboratories statewide. However, we do not have information about the age of the cultures upon their arrival at our laboratory. Additionally, some isolates grown in MGIT show sparse growth due to the high sensitivity of BACTEC, and older isolates on solid media often have few colonies. Despite low growth, these isolates contain more bacilli than clinical samples. Consequently, using the standard 2:1 STR-to-cultured isolate ratio recommended for clinical samples may inhibit the BD MAX qPCR reaction due to potential DNA overload.

To standardize the volume of cultured isolates for testing on the BD MAX, we evaluated an inactivated H37Rv culture grown in MGIT for 28 days, using various culture-to-STR ratios: 100 µL of culture to 2.9 mL of STR, 200 µL of culture to 2.8 mL of STR, and continuing to the maximum of 1 mL of culture to 2 mL of STR. As we obtained valid results for all ratios tested (data not shown), we selected 200 µL of inactivated culture to 2.8 mL of STR (1:15) for subsequent tests, providing a total volume of 3 mL. This ensured the availability of the required 2.5  mL per BD MAX reaction while retaining a sufficient volume of inactivated culture for potential repeat testing.

We also tested whether adding STR would affect BD MAX performance on already inactivated cultures. Three MTBC isolates with known mutations were tested in duplicate, mixing 200 µL of inactivated suspension with either 2.8 mL ultrapure water or STR. Using STR significantly lowered Ct values by 9–10 cycles for all isolates, indicating that STR improves BD MAX assay performance.

### BD MAX MDR-TB testing with NTM and MTBC clinical isolates with known mutations

To assess the specificity of the BD MAX assay on cultured isolates, the following nine species of NTM from our repository were tested: *M. abscessus* subsp. *abscessus*, *M. abscessus* subsp. *bolletii*, *M. abscessus* subsp. *massiliense*, *M. avium*, *M. chelonae*, *M. fortuitum*, *M. intracellulare*, *M. kansasii*, and *M. peregrinum*. A clinical isolate identified as *Nocardia asteroides* was also tested. These isolates were received as part of the laboratory routine and initially identified using the GenoType Mycobacterium CM version 2.0 kit (Bruker-Hain Lifescience). For cases where species-level identification was not achieved with this assay, the in-house PRA-*hsp65* method was employed (21).

To evaluate the BD MAX for drug resistance detection in clinical isolates, 29 MTBC isolates from our repository were used. These isolates had been previously characterized for both phenotypic and molecular resistance to RIF and INH. RIF phenotypic testing was performed using critical concentrations (CC) of 1.0 µg/mL or 0.5 µg/mL (22) (Table 2). Isolate selection was based on mutations identified by either GenoType MTBDR*plus* or DNA sequencing (Table 2).

**TABLE 2:** Clinical isolates of the *Mycobacterium tuberculosis* complex tested retrospectively by the BD MAX MDR-TB assay

N° isolates	Mutations detected by LPA or DNA sequencing			Phenotypic result by BACTEC MGIT 960^*b*^	Comparison between LPA/sequencing and BD MAX	
	Rifampicin, RIF	Isoniazid, INH				
	*rpoB* RRDR	*katG* codon 315^*a*^	*inhA* promoter			
1	F424L^*c*^ + H445N^*d*^	S315T^*d*^	C-15T^*d*^	**RIF (R), INH (R**)^*g*^	RIF and INH concordant	
1	T427A^*e*^	S315T^*d*^	WT	RIF (S), **INH (R**)	RIF and INH concordant	
1	T427T^*f*^ (silent)	WT	WT	RIF (S), INH (S)	RIF resistance detected by BD MAX; INH concordant	
1	L430P^*d*^	WT	C-15T^*d*^	RIF (S), **INH (R**)	RIF and INH concordant	
1	L430R^*e*^	WT	WT	RIF (S), INH (S)	RIF and INH concordant	
1	F433F^*f*^ (silent)	WT	WT	RIF (S), INH (S)	RIF resistance detected by BD MAX; INH concordant	
1	D435F^*d*^	WT (315); 1768delA (K590R_fsTGA624)	WT	**RIF (R), INH (R**)	RIF and INH concordant	
1	D435V^*d*^	S315T^*d*^ + WT	WT	**RIF (R), INH (R**)	RIF concordant; INH resistance detected by BD MAX	
1	D435V^*d*^ + S450L^*d*^ + WT	S315T^*d*^ + WT	WT	**RIF (R), INH (R**)	RIF and INH resistance detected by BD MAX	
1	ΔN437^*f*^	WT (315); Q439R (1316 A > G)	WT	**RIF (R), INH (R**)	RIF and INH concordant	
1	P439L^*e*^	WT	WT	RIF (S), INH (S)	RIF and INH concordant	
1	S441L^*d*^	S315T^*d*^	WT	**RIF (R), INH (R**)	RIF and INH concordant	
1	L443L^*f*^ (silent)	WT	WT	RIF (S), INH (S)	RIF resistance detected by BD MAX; INH concordant	
1	H445D^*d*^	WT (315); P232A (694 C > G)	WT	**RIF (R), INH (R**)	RIF and INH concordant	
1	H445L^*d*^	S315T^*d*^	G-17T^*f*^	**RIF (R), INH (R**)	RIF and INH concordant	
1	H445N^*d*^	WT (315); W300R (898T > C) +C549G (1645T > G)	WT	RIF (S),**INH (R**)	RIF and INH concordant	
1	H445S^*d*^	S315T^*d*^	WT	RIF (S), **INH (R**)	RIF and INH concordant	
1	H445Y^*d*^	S315T^*d*^	WT	**RIF (R), INH (R**)	RIF and INH concordant	
1	S450L^*d*^	S315N^*d*^	C-15T^*d*^	**RIF (R), INH (R**)	RIF and INH concordant	
1	S450W^*d*^	S315N^*d*^	WT	**RIF (R), INH (R**)	RIF and INH concordant (RIF resistance detected after repetition)	
1	L452P^*d*^	S315T^*d*^	WT	RIF (S), **INH (R**)	RIF and INH concordant	
1	L452P^*d*^	WT	T-8C^*c*^ + WT by LPA; WT by sequencing	RIF (S), **INH (R**)	RIF concordant; INH resistance missed by BD MAX even after repetition	
1	L452Q^*f*^	WT	WT	RIF (S), INH (S)	RIF and INH concordant	
1	WT	S315T^*d*^	WT	RIF (S), **INH (R**)	RIF and INH concordant	
1	WT	S315R^*d*^ + WT	WT	RIF (S), **INH (R**)	INH resistance detected by BD MAX	
4	WT	WT	WT	RIF (S), INH (S)	RIF and INH concordant	

^*a*^ Mutations are shown regarding codon 315; for sequenced isolates with mutations in other *katG* regions, the mutation is described.

^*b*^ Underlined are results for isolates tested with the updated RIF critical concentration (CC) of 0.5 µg/mL (the others were tested with RIF CC at 1.0 µg/mL); INH was tested at CC of 0.1 µg/mL; according to the 2023 WHO catalog of mutations (23).

^*c*^ Mutation of uncertain significance.

^*d*^ Mutation associated with drug resistance.

^*e*^ Mutation associated with drug resistance–interim.

^*f*^ Mutation not found on the catalog.

^*g*^ Bold indicates to show resistant results for RIF and/or INH.

### Prospective testing on suggestive-MTBC clinical isolates by the GenoType MTBDR*plus* and BD MAX MDR-TB

#### Sample size calculation

Sensitivity data from our previous studies using the GenoType MTBDR*plus* assay, along with routine testing data from our laboratory, were used to calculate the sample size required for the prospective evaluation of BD MAX performance in our setting.

Applying the sample size calculation formula *N* ≥ *Z*^2^ *P* (1 *− P*) / *D*^2^ (where *N* is the number of drug-resistant samples; *Z* = 1.96 for a 95% CI; *P* is the expected sensitivity of the test [0.89 for INH; 0.93 for RIF]; and *D* is the precision or confidence interval [0.1]) (24), 38 INH-resistant and 25 RIF-resistant isolates were required.

Considering (i) the resistance frequencies to RIF (1.8%) and INH (3.5%) in MTBC isolates tested by MTBDR*plus* in our setting in 2019, (ii) the 3.1% rate of MTBC-negative or invalid tests, and (iii) a 5% loss ratio, we estimated that 1,423 MTBC-suggestive isolates had to be tested to yield 25 RIF-R isolates. Given the higher frequency of INH resistance, this number of samples would also be sufficient to obtain 38 INH-R isolates.

#### Clinical isolates

The mycobacterial isolates are sent to our laboratory for species identification and DST by the TB network laboratories that perform culturing in the state of São Paulo. These isolates are first assessed regarding colony characteristics to presumptively determine whether they belong to the MTBC or are NTMs (25).

All consecutive clinical isolates presumptively identified as MTBC and tested with MTBDR*plus* in our routine between June and August 2023 were prospectively included in this study. Isolates belonged to patients who met the criteria for DST as outlined by the State and National TB Control Programs (26). These criteria are as follows: being a contact of a drug-resistant TB case; having a history of previous TB treatment; being HIV-positive or having other forms of immunosuppression; having a positive smear result at the end of the second month of therapy; belonging to high-risk populations for TB (e.g., healthcare workers, homeless individuals, prisoners, hospitalized patients, and indigenous people); or having a GeneXpert MTB/RIF Ultra RIF-R result.

#### DNA extraction

For DNA extraction, 1 mL of the isolate’s growth in MGIT was distributed into two vials each: one for MTBDR*plus* and the other for BD MAX. For isolates grown on solid media, a loopful of bacterial growth was added to two vials each, with one vial designated for MTBDR*plus* and the other for BD MAX. DNA extraction for MTBDR*plus* was carried out according to the manufacturer’s instructions (27). The BD MAX vials were heat-inactivated and freeze-thawed as described above and kept at −20°C until testing.

#### GenoType MTBDR*plus*

The test was performed according to the manufacturer’s instructions (27). PCR product hybridization was carried out using the GT-Blot 48 (Bruker-Hain Lifescience), which processes up to 48 tests simultaneously. After drying, strips were scanned by the GenoScan system, and results were interpreted by its software based on band patterns (27). Following technician verification, results were released into the laboratory system as per routine.

An inconclusive result was defined when the TUB band, indicating the presence of MTBC, was positive, but the WT bands for the genes were faint, and no mutation (MUT) bands developed due to low bacterial load, impairing the detection of drug resistance. All isolates that yielded inconclusive results were retested with 10 µL of DNA—double the volume used in the initial PCR reaction (5 µL).

Isolates negative for MTBC by MTBDR*plus* were further tested using the Bioline TB Ag MPT64 assay (Abbott, USA)(28). If still negative, they were analyzed with the GenoType Mycobacterium CM v2.0 kit (Bruker-Hain Lifescience). NTM isolates not identified at the species level by this kit were tested using the in-house PRA-*hsp65* method (21). PRA-*hsp65* is a DNA-based method for species identification of mycobacteria that involves PCR amplification of a fragment of the *hsp65* gene, which is found in all species of mycobacteria, followed by restriction enzyme digestion. The resulting fragment patterns, which vary by species, are analyzed via agarose gel electrophoresis and compared with published profiles (21).

#### BD MAX MDR-TB assay

For BD MAX testing, 200 µL of inactivated isolate suspension was mixed with 2.8 mL of STR, shaken vigorously (10 manual up-and-down strokes) to ensure thorough mixing, and incubated for 5 minutes at room temperature. Then, 2.5 mL of the mixture was transferred to the BD MAX sample tube and processed according to the manufacturer’s protocol (10).

BD MAX results included: MTB detected/not detected/low positive (resistance not analyzed in low-positive cases), and RIF/INH resistance detected/not detected. Unreportable indicates MTBC detection without interpretable resistance data, while MTB unresolved means neither MTBC DNA nor sample processing control was detected. Indeterminate signals a system error, and incomplete refers to an unfinished run (10). Isolates with low-positive, unreportable, or indeterminate results were retested using 2.5 mL of a 1:5 proportion (500 µL cultured isolate + 2.5 mL STR). This larger volume was used to rule out the possibility that test failure was due to insufficient DNA quantity.

#### Gene sequencing

Following Pinhata et al. (19), isolates with discordant BD MAX and MTBDR*plus* results, as well as those with inferred mutations by MTBDR*plus*, underwent Sanger sequencing.

The *inhA* promoter (−168 to +80), *katG* gene (−135 to +2202, plus 431 bp downstream), and *rpoB* RRDR region (positions 1184–1533) were amplified using specific primers (29–31). All amplification reactions were performed with GoTaq G2 Flexi DNA polymerase kit and dNTP mix (Promega, USA). Each PCR reaction included 1 × colorless buffer, 200 µM dNTP mix, 1.5 mM MgCl_2_ for *inhA* promoter and *katG*, 1.75 mM MgCl_2_ for *rpoB*, 5 pmol of primers for *inhA* promoter and *katG*, 10 pmol of primers for *rpoB*, 0.75 U of Taq DNA polymerase, approximately 50 ng of DNA template, and nuclease-free water (Qiagen, USA) for a final volume of 25 µL. Cycling conditions included an initial denaturation at 94°C for 5 minutes; 40 cycles of denaturation at 94°C for 1 minute, annealing at 64°C (*inhA*), 62°C (*katG*), or 60°C (*rpoB*) for 1 minute, and extension at 72°C for 1 minute; followed by a final extension at 72°C for 10 minutes.

Amplicons were purified using ExoSAP-it (Affymetrix, USA) and sequenced with ABI 3130 × L Genetic Analyzer and the BigDye Terminator version 3.1 (Applied Biosystems, USA). Sequences were analyzed using BioEdit v7.2.5 and the MUBII-TB-DB and BLAST tools (32).

#### Phenotypic DST by BACTEC MGIT 960

Isolates with mutations not listed on the WHO catalog or with uncertain/interim resistance associations underwent pDST for RIF and/or INH using the BD BACTEC MGIT 960 IR Kit (Becton, Dickinson and Company) at critical concentrations of 0.5 µg/mL (RIF) and 0.1 µg/mL (INH) (33).

pDST was not performed for isolates with WHO-classified mutations associated with drug resistance, as phenotypic confirmation is not required in such cases (23).

#### Data analysis

BD MAX performance for MTBC identification and RIF/INH resistance detection was assessed against MTBDR*plus* and, when needed, gene sequencing. Sensitivity, specificity, and accuracy were calculated (34).

## RESULTS

### Retrospective BD MAX testing with NTM and MTBC clinical isolates

All NTM and *N. asteroides* isolates tested negative for MTBC by the BD MAX assay on the first run. Results for MTBC isolates with known mutations are shown in Table 2. All discordant cases were retested.

BD MAX correctly identified all MTBC isolates on the first run. Of the 29 isolates tested, 23 had *rpoB* mutations, including three with silent mutations (T427T, F433F, and L443L) that BD MAX interpreted as RIF-R, although pDST confirmed them to be susceptible at 1.0 µg/mL of RIF. Nineteen of 20 isolates with non-silent mutations were identified as RIF-R by BD MAX, including two with double mutations (which tested RIF-R by pDST).

pDST showed that nine of the 20 isolates with non-silent *rpoB* mutations were RIF-susceptible (T427A, L430P/R, P439L, H445N, and L452P/Q at 0.5 µg/mL; H445S and L452P at 1.0 µg/mL). One isolate with the high-confidence S450W mutation and phenotypically RIF-R at 1.0 µg/mL was initially missed by BD MAX but detected upon repeat testing. All six isolates with wild-type *rpoB* were correctly interpreted as RIF-susceptible by BD MAX, all of which were phenotypically susceptible at 1.0 µg/mL (Table 2).

For *katG* and *inhA*, BD MAX identified INH resistance in 14 of 15 mutant isolates. The single discordant case showed heteroresistance in *inhA* by LPA (T-8C + WT) but was wild type by sequencing. All 14 wild-type isolates on *katG* 315 and/or *inhA* promoter were correctly reported as INH-susceptible by BD MAX (Table 2).

Interestingly, four isolates were wild-type on both *katG* 315 and *inhA* promoter and were INH-R on pDST. Sequencing of the whole *katG* gene showed that these isolates presented diverse mutations outside the 315 codon (Table 2).

### Prospective BD MAX testing on MTBC-suggestive clinical isolates

We prospectively analyzed 1,440 isolates presumptively identified as MTBC based on colony morphology. Of these, 1,260 (87.5%) were cultured in MGIT and 180 (12.5%) on Löwenstein-Jensen or Ogawa-Kudoh. All isolates were tested with both MTBDR*plus* and BD MAX.

Valid BD MAX results were obtained on the first run for 1,419 isolates (98.5%). Among the 21 (1.5%) invalid isolates, 20 were grown in MGIT and one on solid medium. The initial invalid results included 13 MTBC low positives, four INH unreportable/RIF wild-type, two RIF unreportable/INH wild-type, one RIF unreportable/INH-R, and one indeterminate. Upon retesting, 13 of these 21 initially invalid cases yielded valid results, all concordant with the LPA.

Among the 1,440 isolates, MTBDR*plus* identified 1,409 as MTBC. BD MAX initially detected 1,402 (99.5%) as MTBC-positive, six (0.4%) as negative, and one (0.1%) as indeterminate. After repetition, the indeterminate isolate was subsequently confirmed as MTBC-positive. Of the 31 non-MTBC isolates by MTBDR*plus*, BD MAX correctly identified 30 (96.8%) and misclassified one as MTBC (3.2%). Thus, BD MAX sensitivity, specificity, and accuracy for MTBC detection were 99.6% (1,403/1,409), 96.8% (30/31), and 99.5% (1,433/1,440), respectively.

Regarding MTBC detection, repeat BD MAX testing resolved four of the seven initial discrepancies. Three MTBC-negative results were confirmed as MTBC-positive: two tested MTBC low positive (one of which was inconclusive for drug resistance by LPA, i.e., had low bacterial load), and one was confirmed MTBC-positive. The non-MTBC isolate misclassified as MTBC was corrected (the first result was MTBC low positive). No isolates showed incomplete results by BD MAX. After discrepancy analysis, BD MAX showed 99.8% sensitivity (1,406/1,409), 100% specificity (31/31), and 99.8% accuracy (1,437/1,440) for MTBC detection in cultured isolates.

Final BD MAX and LPA results after retesting the invalid BD MAX isolates are shown in Table 3. BD MAX was concordant in 1,304 (98.9%) of 1,318 wild-type isolates by MTBDR*plus*. Fourteen showed discordance: three reported as RIF-R, one as INH-R, five as non-MTBC, three as MTBC low positive (one with low bacterial load by LPA), and two as INH unreportable/RIF wild-type—both initially MTBC low positive.

**TABLE 3:** Results of GenoType MTBDR*plus* and BD MAX MDR-TB for *Mycobacterium tuberculosis* complex isolates tested prospectively^*e*^

	LPA GenoType MTBDR*plus*					
BD MAX MDR-TB	Wild-type^*a*^	RIF-R	INH-R	MDR	MTBC-positive, resistance inconclusive	Total
Wild-type	1304 > 1307	2^*b*^	1	1	-	1,308
RIF-R	3 > 1	40	-	-	-	43
INH-R	1 > 1	-	30	1^*c*^	-	32
MDR	-	-	-	13	-	13
Non-MTBC	5 > 3	-	-	-	1	6
MTBC low positive	3 > 4	-	-	-	1	4
INH UNR	2^*d*^ > 2^*d*^	-	-	1^*d*^	-	3
Total	1,318	42	31	16	2	1,409

^*a*^ The > symbol separates the results into those obtained after repetition of isolates MTBC low positive, unreportable, and indeterminate isolates by BD MAX (left), and those obtained after BD MAX repetition of discordant isolates between LPA and BD MAX (right).

^*b*^ 1 isolate with a heteroresistant profile in *rpoB* by LPA*.*

^*c*^ RIF unreportable: MTBC DNA detected but RIF resistance metrics not measurable (a single *rpoB* probe failed to give a signal with the remaining *rpoB* probes presenting as wild-type signals).

^*d*^ RIF susceptible.

^*e*^ RIF-R, rifampicin-resistant; INH-R, isoniazid-resistant; MDR, multidrug-resistant; MTBC low positive, *M. tuberculosis* complex DNA detected, resistance metrics not measurable due to low bacterial load; INH UNR, BD MAX error INH resistance unreportable (MTBC DNA detected but INH resistance metrics not measurable; either the *katG* or *inhA* promoter probe failed to give a signal with the other signal presenting as wild-type); MTBC-positive, resistance inconclusive: TUB band positive, but the genes’ bands were faint due to low bacterial load.

Concordance for RIF-R detection was 95.2% (40/42). Of the two discordant cases, one showed low bacterial load on LPA (*rpoB* WT6,7,8 bands absent), and the other had a heteroresistant *rpoB* profile (S450L + WT) and was not retested by BD MAX (Table 3).

Of the 31 INH-R isolates by LPA, 30 (96.8%) were concordant on BD MAX; the single discordant case lacked the *inhA* WT2 band on LPA but was wild-type by BD MAX. Among 16 MDR isolates by LPA, 13 (81.3%) were concordant on BD MAX. Of the three discordant cases, one showed heteroresistance on LPA (*rpoB* WT + D435V and *katG* WT + S315T1) but was wild-type on BD MAX and not retested; another was INH-R/RIF unreportable on BD MAX due to the absence of *rpoB* WT2,3,4 bands and the *katG* S315T1 mutation on LPA; and the third, with absent *rpoB* WT7 and no *katG* bands on LPA, was INH unreportable/RIF wild-type by BD MAX. Two isolates with inconclusive LPA results were MTBC low positive on BD MAX, suggesting low bacterial load (Table 3).

Isolates with persistent discordant results between LPA and BD MAX, even after repeat testing of initially invalid BD MAX cases, were further retested. Among the 14 isolates classified as wild-type by LPA, 11 remained discordant. Repeat testing did not resolve discrepancies for cases identified as RIF-R, INH-R, or MDR by LPA (Table 3).

Final discordant isolates, excluding non-MTBC and MTBC low positives, were analyzed by Sanger sequencing (Table 4). Among LPA wild-type cases, BD MAX correctly identified RIF-R and INH-R heteroresistance, as confirmed by sequencing. Of two INH unreportable/RIF wild-type cases by BD MAX and wild-type by LPA, one was *katG* heteroresistant (missed by LPA), and the other was confirmed wild-type by sequencing, in concordance with LPA.

**TABLE 4:** Gene sequencing results of *Mycobacterium tuberculosis* complex isolates showing resistance discordances between GenoType MTBDR*plus* and BD MAX MDR-TB assays^*c*^^,^^*d*^

MTBDR*plus*result	BD MAX MDR-TB result	Sequencing result
**Wild-type (WT**)	RIF-R (*n* = 1)	*rpoB*_HR D435E^*a*^ + WT(1305 gac>gag + 1305 gac>=)
	INH-R (*n* = 1)	*katG*_HR S315R + WT(945 agc>agg + agc>=)
	INH UNR (*n* = 2)	*katG*_HR S315R + WT(945 agc>agg + agc>=), *inhA*promo_WT
		*katG*_WT, *inhA*promo_WT
**RIF-R** (*rpoB* WT6,WT7,WT8 absent)	Wild-type (*n* = 2)	*rpoB*_WT
**RIF-R** (*rpoB* WT + S450L)		not sequenced
**INH-R** (*katG* WT, *inhA* WT2 absent)	Wild-type (*n* = 1)	*inhA*promo_T-8G^*b*^
**MDR**(*rpoB* WT + D435V; *katG* WT + S315T1)	Wild-type (*n* = 1)	*rpoB*_HR D435V + WT (1304 gac>gtc + gac>=), *katG*_HR S315T + WT (944 agc>aac + 944 agc>=)
**MDR** (*rpoB* WT2,WT3,WT4 absent; *katG* S315T1)	INH-R, RIF UNR (*n* = 1)	*rpoB*_Q432H + F433fs^*a*^(del F433 M434 D435) (1296 caa>cac + del 1297 ttcatggac)
**MDR** (*rpoB* WT7 absent; *katG* locus and WT absent)	INH UNR, RIF susceptible (*n* = 1)	*rpoB*_H445L (1334 cac>ctc); *katG* (whole)_does not align

^*a*^ Mutation associated with drug resistance– interim.

^*b*^ Mutation of uncertain significance.

^*c*^ It was not possible to identify the specific mutation by gene sequencing; HR, heteroresistance.

^*d*^ Only the mutations that differed from those tested during the retrospective phase are highlighted according to the 2023 WHO catalog of mutations (see Table 2).

Of the two isolates classified as RIF-R by LPA but wild-type by BD MAX, sequencing confirmed one as wild-type. This isolate lacked WT6,7,8 bands on LPA, suggesting low bacterial load and a false-resistant result—supported by a RIF-susceptible Xpert Ultra result. The other, showing a heteroresistant profile on LPA, was not sequenced. For the single INH-R discordant isolate, BD MAX missed the *inhA* T-8G mutation (inferred by the absence of the WT2 band on LPA), which was detected by sequencing (Table 4).

Among the discordant MDR isolates, one was wild-type by BD MAX but showed heteroresistance on LPA for *rpoB* and *katG*, confirmed by sequencing. Another was INH-R/RIF unreportable by BD MAX; sequencing revealed an SNP followed by a deletion in *rpoB*, classified as an interim RIF-R mutation. The third, reported as RIF wild-type/INH unreportable by BD MAX, carried the *rpoB* H445L borderline mutation and a *katG* deletion, as indicated by the absence of locus and WT bands on LPA (Table 4).

## DISCUSSION

The BD MAX system represents a new generation of closed, automated assays using medium- to high-throughput platforms, enabling rapid detection of MTBC and resistance to RIF and/or INH. This is the first study evaluating the off-label use of the BD MAX MDR-TB platform in clinical isolates under routine conditions in a high TB burden setting. The results demonstrate the BD MAX system’s robust sensitivity, specificity, and accuracy in both retrospective and prospective analyses.

As a reference laboratory processing exclusively mycobacterial cultures, we have successfully validated BD MAX for clinical isolates, optimizing LoD, STR incubation, and sample-to-STR ratios. Our LoD for MTBC detection in inactivated H37Rv culture was ~34 CFU/mL, while for drug resistance mutations, it was higher at ~340 CFU/mL. This finding is consistent with Beutler et al., who also reported a higher LoD for drug resistance detection compared to MTBC identification (35).

In retrospective testing, BD MAX accurately excluded all NTM and *N. asteroides* isolates on the first run, confirming its high specificity for MTBC. A previous study focusing on BD MAX sensitivity with paucibacillary, smear-negative samples, and samples growing NTM obtained 100% specificity for pulmonary samples growing NTM on culture (13). Given the rise of NTM infections in Brazil (28, 36, 37), BD MAX’s strong ability to differentiate MTBC from NTM is crucial for reliable diagnosis.

Retrospectively tested MTBC isolates with known mutations were all detected by BD MAX, showing high concordance for RIF and INH mutation detection. This aligns with prior studies showing BD MAX’s high sensitivity and specificity for the detection of mutations in various sample types (11, 13, 16, 38).

MTBDR*plus* identifies specific high-confidence mutations in *rpoB*, *katG*, and the *inhA* promoter, while BD MAX only indicates the presence of mutations and the affected gene. This allows rapid results but lacks information on precise nucleotide or codon changes, which may be necessary for detailed resistance profiling or epidemiological studies.

In this study, BD MAX interpreted silent *rpoB* mutations as RIF-R, reflecting its high sensitivity but yielding false RIF-R results, as all three isolates with silent mutations were RIF-susceptible by pDST. Thus, the BD MAX software should be updated to classify silent mutations as RIF-susceptible or to provide users with detailed information on the specific mutations detected, allowing for accurate clinical interpretation. Notably, in our retrospective analysis, BD MAX effectively detected double mutations and heteroresistant isolates, except one with an *inhA* T-8C + WT profile by LPA and INH-R by pDST, which was wild-type by sequencing. This discrepancy likely occurred because LPA was performed on a previous subculture, leading to loss of a minority resistant subpopulation due to genetic heterogeneity or selective growth.

MTBDR*plus*, routinely performed in our laboratory, is more labor-intensive than BD MAX. Furthermore, it requires multiple rooms for different steps of the procedure. DNA extraction and PCR are conducted manually in batches of 47 isolates plus one negative control. Hybridization of the 48 tests is carried out using the GT-Blot 48 equipment, with results analyzed using GenoScan. In our laboratory, the team members who perform the test work four-hour daily shifts. As a result, the entire process typically takes three days: one day for DNA extraction and PCR, another for hybridization, and a third for reading the results after the strips have dried adequately. Interpretation and report release are carried out by team members working eight-hour shifts.

In contrast, BD MAX is significantly simpler to operate. According to our validated protocol, isolates are first inactivated by thermal lysis, then mixed with the STR reagent, shaken vigorously, and incubated for 5 minutes at room temperature. The prepared mixture is then ready to be transferred directly to the BD MAX equipment for automated processing and interpretation. The entire BD MAX workflow can be completed on the same day, as the combined time for inactivation and qPCR is approximately five hours. As results reports are released by our team members working full eight-hour shifts, it is feasible to test and release results for up to 24 isolates per day. While the BD MAX processes fewer samples at once, it offers faster turnaround compared to the three-day MTBDR*plus* process in our workflow.

In addition to testing NTM and mutations of varying frequencies routinely found in MTBC clinical isolates in our setting, we also tested silent *rpoB* mutations to evaluate BD MAX performance. Another key strength of our study is the large number of isolates tested prospectively under real-world routine conditions. This included isolates cultured on solid media or in MGIT with varied ages, as well as those with low bacterial loads or contamination by other microorganisms. Testing a diverse and adequate sample size with various mutations is essential for obtaining precise diagnostic accuracy estimates.

The prospective analysis of 1,440 clinical isolates further validated the BD MAX’s effectiveness in our routine, with 99.6% sensitivity and 96.8% specificity for MTBC detection. Upon repeat testing of the six isolates initially reported as false negative for MTBC by BD MAX, three remained negative, one of which had little DNA in the primary culture. A possible explanation for that difference between LPA and BD MAX results is that the primary cultures were aliquoted into two separate tubes, one for LPA extraction and the other for BD MAX testing, potentially leading to an uneven distribution of bacilli between them.

Most discrepancies were related to low bacterial load or heteroresistance. BD MAX’s performance aligns well with MTBDR*plus*, with the added advantages of automation and reduced hands-on time. In our workflow, inconclusive results obtained by LPA are repeated. Similarly, if BD MAX were used instead of LPA, MTBC low positive, indeterminate, and unreportable results would also undergo repetition before releasing the final report. BD MAX’s ability to resolve discordances and invalid results on retesting highlights its reliability in routine diagnostic settings.

This study showed fewer indeterminate or unreportable results than studies using clinical samples, such as Zimmermann et al. (39), who reported 8.9% indeterminate RIF/INH results. The lower rate is likely due to testing cultured isolates, which have higher bacterial loads than clinical specimens.

Sequencing of discordant isolates tested prospectively revealed that BD MAX detected heteroresistance in two cases missed by LPA: one RIF-R (*rpoB* D435E + WT) and one INH-R (*katG* S315R + WT). However, both assays failed to detect the same *katG* S315R mutation in another heteroresistant isolate, which was INH unreportable by BD MAX and wild-type by LPA. This suggests that heteroresistant subpopulations may have been present below the assays’ detection thresholds. Another isolate, classified as INH unreportable by BD MAX and wild-type by LPA, was confirmed as wild-type by sequencing. Notably, both INH unreportable isolates yielded MTBC low-positive results on the initial BD MAX run, suggesting an association between unreportable resistance results and low bacterial load, as reported by Hofmann-Thiel et al. (13).

BD MAX correctly identified a wild-type isolate misclassified as RIF-R by LPA, likely due to absent WT6,7,8 bands—commonly associated with low bacterial load. This underscores BD MAX’s lower LoD compared to LPA. As reported by manufacturers, BD MAX’s LoD is 0.25 CFU/mL for MTBC detection in sputum specimens (10), while LPA’s LoD is much higher at 160 CFU/mL (27).

In prospective testing, BD MAX missed specific mutations detected by LPA, including an *inhA* T-8G mutation and a heteroresistant MDR isolate with *rpoB* D435V + WT and *katG* S315T + WT—despite the patient having a RIF-susceptible Xpert Ultra result, reflecting challenges in heteroresistance detection. BD MAX also failed to resolve specific inferred resistance profiles, such as an *rpoB* Q432H + F433fs double mutation (RIF unreportable) and an isolate with *rpoB* H445L and *katG* deletion (RIF wild-type/INH unreportable). These cases underscore BD MAX’s limitations in mutation coverage and interpreting complex resistance patterns.

Taken together, our findings indicate that while BD MAX offers rapid and automated detection of MTBC and associated drug resistance, its performance may be affected by factors, such as low bacterial load, heteroresistance, and limited mutation coverage, which can lead to unreportable or indeterminate results. Complementary testing with pDST or sequencing may still be necessary in selected cases to ensure accurate resistance profiling and guide appropriate clinical management.

Our study has some limitations. Mycobacterial isolates tested prospectively were received from many TB laboratories and varied in age and bacterial load, which may have affected the homogeneity of testing conditions. Although cultured isolates-to-STR volume ratios were standardized, variability in isolates’ conditions might still influence results. Furthermore, gene sequencing by Sanger could not specifically identify the mutation present in the MDR isolate lacking the *katG* locus and WT bands on LPA. In this case, whole-genome sequencing would elucidate the specific mutation. Additionally, the lack of pDST for all isolates harboring mutations is a limitation, as it could detect the effect of compensatory mutations that may be present elsewhere in the MTBC genome.

In conclusion, the BD MAX platform proves to be a valuable tool for TB diagnostics, particularly in high-burden settings, offering reliable detection of MTBC and resistance profiling in cultured isolates. Its strong performance supports integration into the routine diagnostics of high-volume reference laboratories, with sequencing or pDST recommended as a confirmatory method for complex or inconclusive cases.

## Data Availability

The DNA sequences of all the sequenced isolates were deposited in GenBank (http://www.ncbi.nlm.nih.gov/) under the following identification: PRJNA888434.
